# Menstrual blood-derived stem cells: toward therapeutic mechanisms, novel strategies, and future perspectives in the treatment of diseases

**DOI:** 10.1186/s13287-019-1503-7

**Published:** 2019-12-21

**Authors:** Lijun Chen, Jingjing Qu, Tianli Cheng, Xin Chen, Charlie Xiang

**Affiliations:** 1 State Key Laboratory for Diagnosis and Treatment of Infectious Diseases, Collaborative Innovation Center for Diagnosis and Treatment of Infectious Diseases, The First Affiliated Hospital, College of Medicine, Zhejiang University, Hangzhou, 310003 Zhejiang People’s Republic of China; 2 Lung Cancer and Gastroenterology Department, Hunan Cancer Hospital, Affiliated Tumor Hospital of Xiangya Medical, School of Central South University, Changsha, 410008 People’s Republic of China; 3 Stowers Institute for Medical Research, 1000 E 50th Street, Kansas City, MO 64110 USA; 4 Department of Respiratory Disease, Thoracic Disease Centre, The First Affiliated Hospital, College of Medicine,, Zhejiang University, Hangzhou, 310003 Zhejiang People’s Republic of China; 5 Thoracic Medicine Department 1, Hunan Cancer Hospital, Affiliated Tumor Hospital of Xiangya Medical, School of Central South University, Changsha, 410008 People’s Republic of China

**Keywords:** Menstrual blood-derived stem cell, Therapeutic strategy, Mesenchymal stem cell, Cellular therapy, Perspective

## Abstract

Menstrual blood-derived stem cells (MenSCs) have great potential in the treatment of various diseases. As a novel type of mesenchymal stem cells (MSCs), MenSCs have attracted more interest due to their therapeutic effects in both animal models and clinical trials. Here, we described the differentiation, immunomodulation, paracrine, homing, and engraftment mechanisms of MenSCs. These include differentiation into targeting cells, immunomodulation with various immune cells, the paracrine effect on secreting cytokines, and homing and engraftment into injured sites. To better conduct MenSC-based therapy, some novel hotspots were proposed such as CRISPR (clustered regularly interspaced short palindromic repeats)/cas9-mediated gene modification, exosomes for cell-free therapy, single-cell RNA sequence for precision medicine, engineered MenSC-based therapy for the delivery platform, and stem cell niches for improving microenvironment. Subsequently, current challenges were elaborated on, with regard to age of donor, dose of MenSCs, transplantation route, and monitoring time. The management of clinical research with respect to MenSC-based therapy in diseases will become more normative and strict. Thus, a more comprehensive horizon should be considered that includes a combination of traditional solutions and novel strategies. In summary, MenSC-based treatment has a great potential in treating diseases through diverse strategies, and more therapeutic mechanisms and novel strategies need to be elucidated for future regenerative medicine and clinical applications.

## Background

Mesenchymal stem cells (MSCs), also termed as mesenchymal stromal cells, are pluripotent progenitor cells with self-renewal ability and differentiating potential [1, 2]. The fundamental properties of MSCs according to the International Society for Cellular Therapy should be addressed with respect to the following three aspects: (1) MSCs should be mechanically adherent in plastic and can be passaged in standard culture media; (2) MSCs must be positive for expressing CD73, CD105, and CD90 and they should be negative for the expression of CD34, CD45, CD11b or CD14, CD19, or CD79α, and human leukocyte antigen (HLA)-DR surface marker molecules; (3) MSCs should differentiate into a variety of cells, including osteoblast, chondrocyte, and adipocyte in vitro [3]. MSCs can be acquired from numerous tissues, including bone marrow (BM) [4], adipose tissue (AD) [5], umbilical cord (UC) [6], placenta [7], endometrium [8], amniotic membrane/fluid [9, 10], synovial membrane/fluid [11], and other solid organs (such as muscle, liver, spleen, lung, kidney, pancreas, and thymus) [12]. Although bone marrow-derived mesenchymal stem/stromal cells (BM-MSCs) have been predominantly studied, separating an adequate amount of BM-MSCs remains a limiting factor owing to the requirements of invasive procedures and donor expansion [13–15]. With the development of multilevel and precision medicine, even the same disease will need more treatment modalities rather than conventional therapies for serving patients. Furthermore, with the exception of some common sources of MSCs (including BM-MSCs, adipose tissue (AD)-MSCs, and umbilical cord (UC)-MSCs), other sources of MSCs should be brought to the forefront since these MSCs probably possess their own merit for a more appropriate therapeutic effect. Therefore, many researchers have focused on exploring novel sources of MSCs.

In 2007, Meng et al. first identified a novel source of stem cells from human menstrual fluid, called endometrial regenerative cells [16]. Subsequently, these cells were named menstrual blood-derived cells, menstrual blood stem cells, menstrual blood-derived stromal stem cells, menstrual blood-derived mesenchymal stem cells, and many more. Menstrual blood-derived stem cells (MenSCs) are the term used throughout this review, which is consistent with our previous studies [17, 18]. Over the last 12 years, researchers have gained more interest in MenSCs due to their advantages of being an abundant and continuous source, procurement via non-invasive procedure, high proliferative rate, low immunogenicity, and lack of ethical issues when compared with other source of MSCs [19–21]. More importantly, MenSCs could be stably amplified for at least 20 passages without mutations or visible abnormalities in vitro [16, 22, 23]. On the basis of these advantages, more researchers focus on therapeutic potentials and underlying mechanisms of MenSCs in treating a series of diseases both in vivo and in vitro. In this review, we will systematically analyze the therapeutic mechanisms and innovative strategies of MenSCs with regard to treating diseases. In addition, we will highlight promising perspectives of MenSC-based therapies in medical research.

## Definition and identity of MenSCs

Before 2007, researchers focused mainly on endometrial stem cells and they did not consider the practical importance of MenSCs [24]. In 2007, Meng et al. and Cui et al. identified MenSCs and further explored their therapeutic potential [16, 25]. Despite the fact that endometrial stem cells and MenSCs shared similar phenotypes and characteristics, the therapeutic effects and mechanisms of both these cells were distinctive [19, 26]. Therefore, MenSCs should not be considered a kind of endometrial stem cells as the two are different but related cell types. According to immunophenotype analysis, MenSCs do not express hematopoietic stem cell markers (including CD19, CD 34, CD45, and CD133) and HLA-DR, and they express classical MSC markers (such as CD29, CD73, CD90, and CD105) and some other surface molecules (such as CD9, CD44, CD166, and HLA-ABC) [19, 20]. Interestingly, MenSCs specifically possess the embryonic stem cells marker, octamer binding transcription factor 4 (OCT-4) [22]. However, expression of the c-kit proto-oncogene (c-kit)/CD117 and stage-specific embryonic antigen-4 (SSEA-4) have to be further clarified due to controversial reports [17, 19]. The definition and identity of MenSCs should be described as follows: (1) the source should be obtained from the menstrual fluid rather than the endometrium; (2) these cells express surface markers CD9, CD29, CD44, CD73, CD90, CD105, CD166, HLA-ABC, and OCT-4, and they are negative for the expression of CD19, CD 34, CD45, CD133, and HLA-DR; (3) MenSCs can be cultured and passaged in plastic-adherent containers and effectively differentiate into osteocytes, adipocytes, and chondrocytes under appropriate conditions.

## Therapeutic mechanisms of MenSCs

**Fig. 1:**
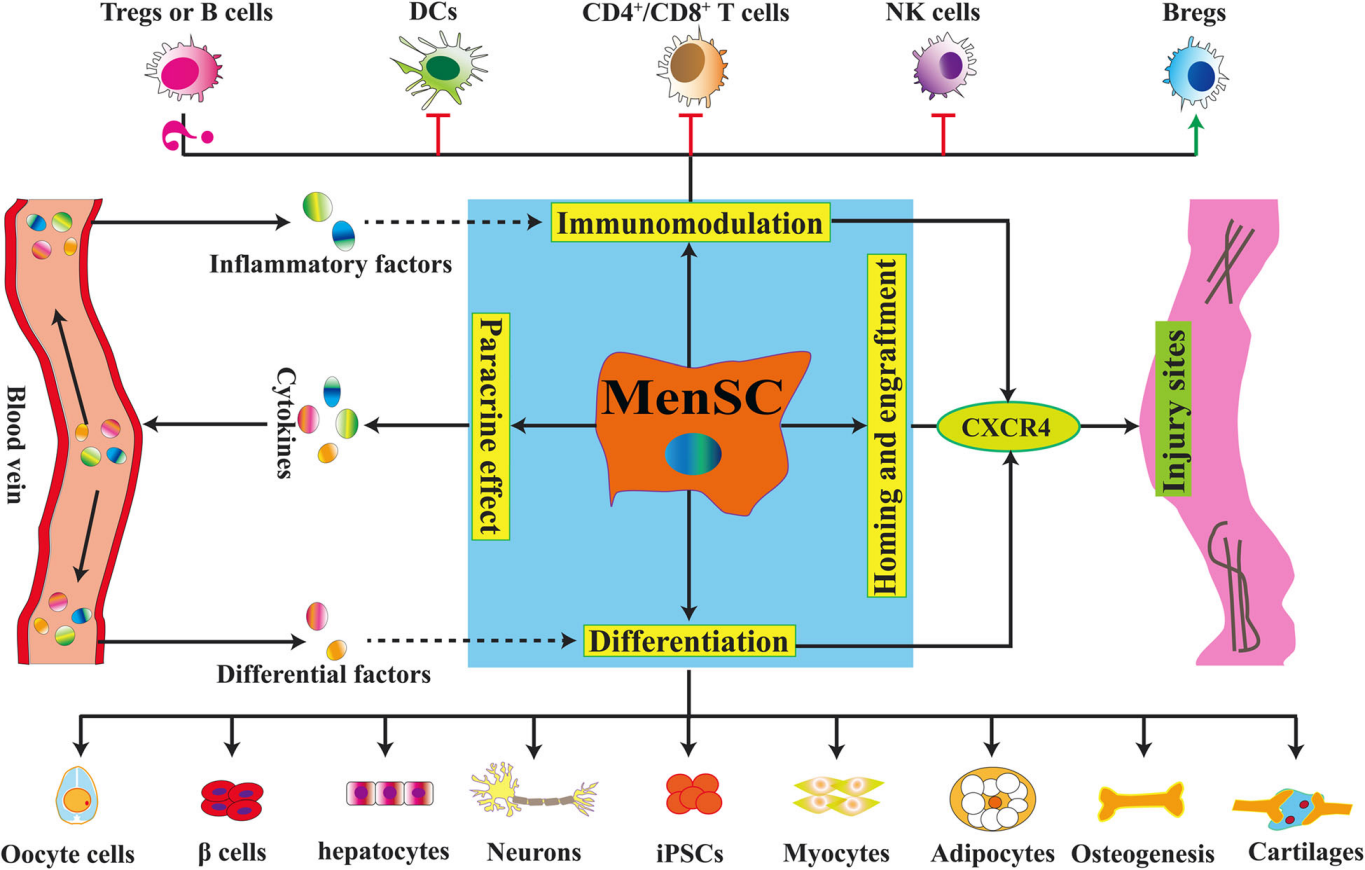
MenSCs exert therapeutic effects mainly via following mechanisms: (1) differentiation into targeting cells, such as cartilaginous, adipocytic, osteogenic, cardiogenic, muscle, neurogenic, oocyte-like, iPSCs, myocytic, granulosa, and hepatic tissues; (2) immunomodulation interacting with various immune cells, such as inhibiting the proliferation of T lymphocyte cell (T cell), natural killer (NK) cell, and dendritic cell (DC), and promoting the production of regulatory B (Breg) cell; (3) paracrine effect secreting a variety of cytokines, such as VEGF, BDNF, NT-3, IL-4, TGF-β 2, EGF, PDGF, NO, HIF-1α, MMP-3, MMP-10, IL-6, MCP-1, HGF, IL-8, GRO, OPG, angiopoietin, elastin, thrombospondin-1, SDF-1, and IGF-1. MenSCs secrete these cytokines through the blood vein to give rise to inflammatory factors, and they interact with the immunomodulation. Similarly, some differential factors also released by paracrine effect through the blood vein to exert the role of differentiation; and (4) homing and engraftment targeting injured sites by some chemokine receptors (such as CXCR4). Green arrows mean positive role, Red T-shapes mean negative role

The therapeutic potential for tissue repairs of MSCs has been extensively studied [27–31]. MenSCs have similar functions and mechanisms consistent with common sources of MSCs (including BM-MSCs, AD-MSCs, and UC-MSCs). Based on current studies, MenSCs exert therapeutic effects mainly through the following mechanisms: differentiation into targeting exogenous cells, immunomodulation interacting with various immune cells, effective secretion of a series of paracrine cytokines, and homing and engraftment targeting into injured sites. An overview of possible therapeutic mechanisms of MenSCs is depicted in Fig. 1.

### Differentiation of MenSCs

The idea of using stem cells capable of differentiation to treat diseases depends on a simple assumption that supplementation with transplanting stem cells can differentiate into desired cells to replace diseased tissues and improve local injuries [32]. Previous studies confirmed that MenSCs possessed remarkable differentiation capacity into various cells, including cartilaginous, adipocytic, osteogenic, cardiogenic, cardiomyocytic, endothelial, muscle, neurogenic, glial-like, oocyte-like, respiratory epithelial, myocytic, hepatic, granulosa, and pancreatic tissues [23, 33, 34]. Additionally, MenSCs could also be an alternative source for producing that induce the development of induced pluripotent stem cells (iPSCs) [35], which are universal cells inducing into almost all cell types. Thus, it is promising to utilize the differentiating potential of MenSCs in treating diseases.

Currently, various methods of MenSC differentiation have been established by different groups in treating corresponding diseases. Cui et al. showed that MenSCs were differentiated into dystrophied myocytes in vitro, and further indicated that MenSCs improved Duchenne muscular dystrophy (DMD) in a mouse model via its differentiating capacity to supplement myocytes in vivo [25]. Liu et al. confirmed that MenSCs had the ability to differentiate into ovarian tissue-like cells in vitro and showed that transplantation of MenSCs could improve ovarian repair in premature ovarian failure (POF) mice through its differentiating potency [36]. Moreover, Lai et al. further demonstrated that MenSCs could differentiate into oocyte-like cells using appropriate media, and induced cells also expressed oocyte-like cell markers, such as the luteinizing hormone receptor and follicle stimulating hormone receptor [37]. Recently, Zheng et al. showed that MenSCs effectively differentiated into endometrial cells in vitro when grown in a medium containing transforming growth factor-β (TGF-β)-1, 17β-estradiol valerate, platelet-derived growth factor (PDGF)-BB, and epidermal growth factor (EGF), and that transplanted MenSCs could rebuild the endometrial structure in a gonadotropin-releasing hormone agonist-induced intrauterine adhesion (IUA) mouse model [38]. Interestingly, our group and Khanjani et al. collectively reported MenSCs could effectively differentiate into functional hepatocyte-like cells in a similar medium containing hepatocyte growth factor (HGF), fibroblast growth factor-4 (FGF-4), EGF, and oncostain M (OSM) in vitro [39, 40]. These differentiated cells also expressed hepatocyte-specific markers, such as albumin (ALB), α-fetoprotein (AFP), cytokeratin-18/19 (CK-18/19), and cytochrome P450 1A1/3A4 (CYP 1A1/3A4). According to some functional examinations, differentiated hepatocyte-like cells were further demonstrated to possess hepatocyte-specific properties including ALB secretion, cytochrome P450 expression, urea synthesis, glycogen storage, and indocyanine green uptake. In addition, our team proved the therapeutic effect of MenSCs in improving type 1 diabetes mellitus (T1DM) in mice. Our previous study showed that MenSCs induced β-cell regeneration and enhanced the number of β-cells by facilitating endogenous progenitor cell differentiation into β-cells after MenSC transplantation in improving T1DM mice [22]. Interestingly, Azedi et al. showed MenSCs could be differentiated into glial-like cells by measuring upregulate levels of glial fibrillary acidic protein, oligosaccharide-2, and myelin basic protein and by downregulating the expression of Nestin protein in vitro [41], which could be a foundation for the treatment of a range of neurological diseases using MenSC. Indisputably, even MenSCs can trans-differentiate in vitro and in vivo into multiple cells, more diseases need to be explored, depending on the mechanism of MenSCs differentiation.

### Immunomodulation of MenSCs

The contribution of MSCs to immunomodulation has been extensively illuminated, and MSCs can modulate innate immune responses and adaptive immune responses via interaction with various immune cells, including inhibiting the proliferation of T cells, B cells, dendritic cells (DCs), and natural killer (NK) cells and promoting regulatory T cells (Tregs) [42, 43]. Currently, great progress has been made in explaining the immunological properties of BM-MSCs; however, studies on immunomodulation of MenSCs are relatively rare in comparison with BM-MSCs. Although some similarities exist between BM-MSCs and MenSCs, there are still some distinctions in extrapolation of functional or regenerative properties. Moreover, the specific functional property is a vital indicator to reach clinical application. Recently, Cuenca et al. revealed that MenSCs had more powerful immunomodulatory properties shown by inhibition of proliferation of T cells via mimic cutaneous damage when compared to UC-MSCs [44]. Moreover, Luz-Crawford et al. found that MenSCs possessed a lower suppressive effect than BM-MSCs in inhibiting the proliferation of T cells. Upon further assessment, MenSCs increased the survival of xeno-graft versus host disease (GVHD) in mice by limiting the proliferation of CD4^+^IFN-γ^+^ or CD8^+^IFN-γ^+^ T cells exerting an immunosuppressive function. Additionally, they found some cytokines such as prostaglandin E-2 (PGE-2), programmed cell death-ligand 1 (PDL-1), indoleamine 2,3 dioxygenase (IDO), and activin A that played a vital role [45]. Bozorgmehr et al. demonstrated that MenSCs modulated the immunomodulatory effect by blocking the generation and maturation of DCs, and secreting interleukin (IL)-6 and IL-10 by acting as an important mediator [46]. Wang et al. indicated that MenSC treatment lowered the survival of mice undergoing experimental colitis. They also found fewer pathological changes in colon tissue that were regulated by increasing the production of regulatory B cells (Bregs) and the expression of IL-10 and cxc chemokin receptor 4 (CXCR4), via immunomodulation of MenSCs [47, 48]. They further proved that this effect was mainly contributed to the increase in expression of B-cell lymphoma-2 (Bcl-2), HGF, and matrix metalloproteinases (MMP)-9, ameliorating idiopathic pulmonary fibrosis [49]. Recently, Shokri et al. demonstrated that MenSCs together with IFN-γ lowered the inhibitory role of MenSCs on NK cell cytotoxicity against K562 target cells. In addition, MenSCs were significantly suppressed by NK cell-mediated lysis [50]. Notably, MenSCs were capable of suppressing immune cells by amplification of pro-inflammatory signals [19, 51].

Therefore, MenSCs modulate immune-modulatory effects via promoting Bregs and inhibiting T cells, DCs, and NK cells (Fig. 1). Compare with BM-MSCs, the role of how MenSCs interact with B cells and Tregs is still unknown; it should be clarified in future research. Although the broader or even the specific mechanisms with immunoregulatory properties of MenSCs are not fully elucidated, the mechanism of the immunomodulation is vital with regard to MenSC-based therapy both in animal model and clinical research.

### Paracrine effect of MenSCs

Although it is initially assumed that MenSCs regenerate tissue by differentiating into desired cells for disease treatment, several researchers have proved that MenSCs repair damaged tissues and promote functional recovery through paracrine effects rather than cell differentiation. Thus, soluble factors secreted by MenSCs play a crucial role in improving tissue regeneration and protecting target cells from cell apoptosis or further injury.

Borlongan et al. observed that transplantation of MenSCs ameliorated ischemic stroke in oxygen glucose deprivation (OGD)-induced rats in vivo by improving behavioral and histological disorders. They also found that MenSCs inhibited cell death of primary neurons in rats by secreting a few paracrine factors, including brain-derived neurotrophic factor (BDNF), vascular endothelial growth factor (VEGF), and neurotrophin 3 (NT-3) in vitro [52]. Additionally, Wu et al. found transplantation of MenSCs improved functional repair of spinal cord injury in rats via upregulation of BDNF [53]. Murphy et al. showed that MenSCs effectively improved critical limb ischemia (CLI) in mice by expressing paracrine factors of IL-4, hypoxia inducible factor-1 alpha (HIF-1α), MMP-3, and MMP-10 [54]. Jiang et al. further demonstrated that administration of MenSCs visibly reduced cell apoptosis and promoted cell proliferation in rats with myocardial infarction (MI). This was primarily regulated by secreted cytokines including PDGF, EGF, nitric oxide (NO), and TGF-β2 in order to activate AKT/extracellular signal-regulated kinases 1 and 2 (ERK 1/2)/signal transducers and activator of transcription 3 (STAT 3) signaling pathway [55]. Recently, our group demonstrated that MenSCs possessed therapeutic effects for improving liver function and reducing collagen deposition post cell transplantation in CCl_4_-induced liver fibrotic mice in vivo [18]. Further investigation indicated that the major contributor for inhibiting activated hepatic stellate cell was the secretion of paracrine cytokines, such as monocyte chemoattractant protein-1 (MCP-1), growth-related oncogene (GRO), IL-6, HGF, osteoprotegerin (OPG), and IL-8 in vitro. More recently, Cuenca et al. demonstrated MenSCs have vital role in improving wound repair in mice by secreting cytokines, such as PDGF, angiopoietin, MMP-3, MMP-10, and elastin [44]. Furthermore, Zhang et al. proved that administration of MenSCs relieved IUA in intrauterine-injured rats in vivo through secretory cytokines of thrombospondin-1, stromal cell-derived factor-1 (SDF-1), and insulin-like growth factor (IGF)-1 [56]. Owing to the above, MenSCs can play a vital role in the treatment of various diseases through paracrine effects, and the soluble cytokines responsible for this were VEGF, BDNF, NT-3, IL-4, TGF-β 2, EGF, PDGF, NO, HIF-1α, MMP-3, MMP-10, IL-6, MCP-1, HGF, IL-8, GRO, OPG, angiopoietin, elastin, thrombospondin-1, SDF-1, and IGF-1. More secretory factors should be explored in future research.

### Homing and engraftment of MenSCs

MSCs communicate with other cells in an organism and accordingly respond to damaged cells, and this is referred to as cell homing and engraftment [57]. Similarly, MenSCs also have the ability to migrate into injury sites to facilitate injury repair acting. According to our previous studies, damaged tissues expressed multiple receptors and ligands (such as CXCR4 and SDF-1) in order to facilitate migration [58, 59]. In addition, chemokines are released to form a gradient, which could guarantee effective access of MenSCs into injury sites.

Alcayaga-Miranda et al. discovered that MenSCs possessed a superior capacity of the migration when compared with BM-MSCs, and the migratory properties were predominantly mediated by some integrins, selectins, and chemokine receptors [51]. Furthermore, Zhu et al. proved that MenSCs notably increased the proliferation and migration capacity of impaired endometrial stromal cells in vitro, which provided a vital foundation for treating endometrial-related injury [60]. Interestingly, Wang et al. demonstrated that transplantation of MenSCs effectively ameliorated cisplatin-induced POF in mice, and the preferential contributor was directive migration into ovarian interstices to regulate the microenvironment of the organism [61]. Therefore, homing and engraftment contributed to MenSC participation in tissue regeneration and continuous delivery of signaling molecules to targeted areas. Although current knowledge regarding homing and engraftment of MenSCs is relatively insufficient, more molecules (other than CXCR4) involved in this process will be brought to light in the near future.

## Novel strategies of MenSCs in treating diseases

**Fig. 2:**
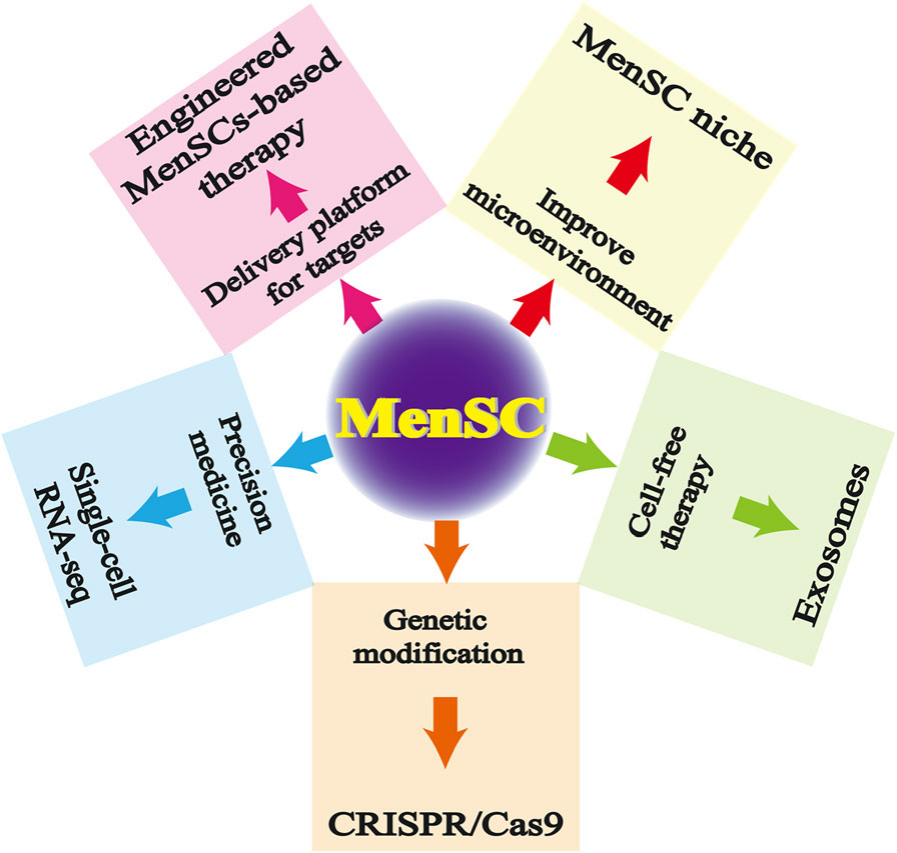
Some novel strategies of MenSCs with regard to treating various diseases, providing a more comprehensive and effective method in MenSC-based therapy. The novel strategies of MenSCs are as follows: CRISPR/cas9 for gene modification, exosomes for cell-free therapy, single-cell RNA-seq for precision medicine, engineered MenSC-based therapy for delivery platform to the targeting site, and niche cells for improving microenvironment

Currently, the therapeutic use of MenSCs remains unclear in clinical trials. No more than 10 clinical trials are registered by recording “menstrual blood stem/stromal cells or menstrual blood-derived cells” (www.clinicaltrials.gov/). In fact, the therapeutic effects of MenSCs have been reported for the treatment of various diseases [17], and interest is rapidly growing in recent years [21, 34, 62, 63]. Therefore, novel strategies (Fig. 2) using MenSCs for the treatment with regard to treating various diseases are extremely necessary and will provide more comprehensive and effective ways for MenSC-based therapy.

### CRISPR/Cas9

Genome editing has great potential in functional genomics, transgenic animal, and gene therapies and is been widely used globally. Genomic editing is based on programmable and highly specific nucleases that produces site-specific cleavage and subsequently induces cellular DNA repair [64]. CRISPR/Cas9 is a recently discovered novel genome editing technology that is widely used in genetic modification, transcriptional regulation, and gene therapy [65]. Sun et al. proved that CRISPR/Cas9 can be applied to many biological organisms with the deep connection of genetically engineered stem cells, factors, and diseases [66]. In addition, Zhang et al. focused on the basic biology of the CRISPR/Cas9 system for application in current stem cell research and discussed future development and prospects of CRISPR/Cas9 in combination with stem cells in biomedical research and regenerative medicine [67]. With the development of MenSC-based therapy, CRISPR/Cas9 can also be used in precise and complex genetic manipulation to enhance the capacity of MenSCs with reprogramming and differentiation in disease models. Recently, Deryabin et al. found that genetic manipulations of MenSCs were effective via CRISPR/Cas9 technology in targeting plasminogen activator inhibitor-1 [68]. Although the initial study is late, there is no doubt that CRISPR/Cas9 will be a promising and highly specific method for genetic modification of MenSCs in treating various diseases.

### Exosomes

Exosomes are small vesicles (30–100 nm in diameter) released by different types of cells containing some microRNA/lncRNA, secreted proteins, lipidosomes, and nucleic acid variants, which regulate animal physiology in vivo and mediate cellular signaling pathways in vitro [69, 70]. The small size and relative mobility of exosomes provide a stable mechanism of biological signals for delivering biomolecules throughout the organism. Several studies have showed that exosomes secreted by MenSCs served as a convincing new type of cell-free treatment, and this approach has no ethical issues and immune rejection is not a concern. Notably, our group found that MenSC-derived exosomes reduced mortality in D-galactosamine/lipopolysaccharide-induced acute liver failure (ALF) in mice by lowering hepatocyte apoptosis to improve liver function [71]. In the same year, Wang et al. found that MenSCs have a better cardioprotective effect than BM-MSCs/AD-MSCs. They further demonstrated that microRNA (miR)-21 of MenSC exosomes kept its cytoprotective function by targeting the homolog (PTEN) and AKT/PKB (protein kinase B) signaling pathway [72]. Recently, Rosenberger et al. indicated that MenSC-derived exosomes showed an inhibitory role on endothelial cells to impede angiogenesis and growth of tumor cells [73]. Another study demonstrated that exosomes of MenSCs improved cutaneous non-healing wounds in diabetic murine models via upregulating NF-κB (nuclear factor-κB) p65 element and further activating the NF-κB signaling pathway [74]. Although recent advances have been reported on exosomes of MenSCs in treating some diseases, greater therapeutic potential of MenSC-derived exosome evacuation is needed in the future.

### Single-cell RNA sequence

Precision medicine is a personalized treatment strategy that takes into account individual differences that can play an important role in prevention, diagnosis, screening, and effective treatment to provide a strong framework for facilitating medical development [75]. Accurate identification has the potential to make interventions more specific and thus prevent disease risk, which is an important goal of precision medicine [76]. Single-cell RNA sequence (RNA-seq) enables unbiased, high-throughput, and high-resolution transcriptional analysis of individual cells. This method provides an additional dimension to transcriptome information, which describes the total number of cells in cell populations. When compared with traditional sequencing methods, it provides new biological technology for tissue composition, transcriptional dynamics, and intergenic regulation [77]. With the rapid development of MenSC-based therapy in recent years, organic compounds can be further engineered to mimic disease-related genetic and epigenetic states, combining state-of-the-art genome editing tools. Therefore, single-cell RNA-seq can provide a unique system for the development of biomedical research as well as drug models for personalized medicine. Recently, our group utilized RNA-Seq to explore the genome-wide type of DNA methylation in hepatocellular carcinoma (HCC) after MenSC treatment [78]. Without a doubt, single-cell RNA-seq can directly detect transcriptome information in single cells of MenSCs. We can use this information directly for precisely targeting specific genes or proteins in the treatment of various diseases in the future.

### MenSC niche

Stem cell function is strictly controlled by the microenvironment of niches and inherent programs. Stem cell niches are defined as cellular and molecular microenvironments that regulate stem cell function [79, 80]. Studies have shown that MSCs themselves were critical for the formation of niches, which help to elucidate the maintenance and differentiation of hematopoietic stem cells [81]. However, the knowledge of MSC niche is still a mystery, and MenSC niche is also difficult to investigate. Recently, Baryawno et al. provided a systematic and comprehensive single-cell RNA-seq for mapping the bone marrow niche. This will be a promising way to further identify niche cells of MSCs with MSC-descendent osteolineage cells [82]. Currently, MSC/MenSC niche (although not yet investigated) that includes several types of cells is a complex microenvironment that is regulated by some complicated interactions, such as cell-to-cell, cell-matrix, and signaling molecules, that activate transcription programs or specific cellular pathways depending on disease or tissue injury. Thus, improving MenSC microenvironment and identifying these niche cells may be a novel method for treating diseases in future.

### Engineered MenSC-based therapy

The anti-tumor efficacy of oncolytic adenovirus (OAdv) is restricted by a number of factors including liver sequestration, blood interactions, immune system elimination, and physical disorders of the tumor. These OAdvs display potential in cancer therapy owing to their capacity to undergo continuous replication and functional induction of tumor cell death in organisms. Moreno et al. showed that MenSCs were promising delivery platforms to target sites with OAdv in the tumor via rapidly viral replication and induction the death of tumor cells [83]. In addition, they found that allogeneic peripheral blood mononuclear cells and OAdv-transduced MenSCs exert a synergistic effect to strengthen antitumor function both in vivo and in vitro [62]. Recently, our group also demonstrated that MenSCs could act as a delivery platform for drug targeting using CRAd5/F11-OAdv-transduced MenSCs for a colorectal cancer (CRC) model in mice [58]. Although the use of MenSCs is still in its infancy, we believe that engineered MenSC-based therapy will be a valuable tool for treating tumor-related diseases.

## Future perspectives of MenSCs in treating diseases

Although the clinical research of BM-MSCs is rapid [84–87], there is still little information on MenSCs in clinical reports. Zhong et al. described the feasibility of allogeneic transplantation of MenSCs in four patients with multiple sclerosis, and no side effects were observed at the 1-year follow-up in this clinical study [88]. Tan et al. reported that autologous transplantation of MenSCs increased the thickness of the endometrium in five women suffering with severe Asherman’s syndrome (AS) [89]. Currently, MenSCs have demonstrated valuable effectiveness in treating a variety of diseases, including stroke, T1DM, acute and chronic liver diseases, acute lung injury, DMD, epithelial ovarian cancer, POF, AS, CRC, Alzheimer’s disease, cardiac diseases, cutaneous wounds, endometriosis, and neurodegenerative diseases [17, 20, 34]. No side effects have been reported with regard to production of tumor post MenSC transplant [16, 22]. Although both clinical data indicated that MenSCs had a therapeutic effect in treating multiple sclerosis and AS, there are still many challenges that need to be addressed prior to MenSC application as a routine choice.

MenSC-based treatment requires further research and verification, including age of donor, appropriate dose, selection of optimal transplantation routes, systematic study of various diseases, and long-term monitoring of MenSCs [34, 90]. A series of literatures are listed in Table 1. First, MenSCs are collected from a donor between the ages of 18 and 45, and many literatures lack the basic information. It is necessary to systematically assess the differences in various stages of MenSCs and then determine which stage is more suitable for treating various diseases. Second, although MenSCs have positive effects on animal models for basic human diseases in clinical research, dosage is largely different in mice (from 1 × 10^4^ to 2 × 10^7^ cells) [25, 36], rats (from 1 × 10^5^ to 3 × 10^6^ cells) [53, 100], and humans (from 1 × 10^6^ to 6 × 10^6^ cells) [88, 89]. The dosage of MenSCs must be further investigated and approved values established for future clinical use. Third, there are many methods of MenSC transplantation including intratumoral, intrathecal, intracerebral, intramyocardial, muscle, intraperitoneal, intravenous, tail vein, subcutaneous, orthopotic, intradermal, thoracic, aorta, hippocampus, and axillary subcutaneous injections. However, only a few researchers have focused on the best method for MenSC transplantation that allows improvement in disease treatment. Therefore, the manner in which MenSCs are injected should be uniformed and agreed upon for further studies of the same disease. It has been demonstrated that MenSC transplantation was safe when evaluating transplanted cells in both animal models and clinical trials [38, 60, 74, 90, 102]. According to Table 1, the collection time post MenSC administration is different (from 2 to 40 days) in pre-clinical research in vivo. There are very few studies concerning the long-term safety or sustained therapeutic effect. Therefore, the survival time of MenSCs in foreign bodies is uncertain, and there are no data that ensure their long-term safety in an extraneous host. Even though therapeutic effects have been proved in the treatment of various diseases, the detailed mechanisms and underlying signaling pathways with regard to these therapeutic effects remain unknown. In addition, Ren et al. found that MenSCs were easily contaminated by multifarious bacteria during the isolated period [95]. Women with premature ovarian insufficiency (POI) are amenorrheic, thereby making collection and usage of autologous MenSCs for themselves impossible [97]. Therefore, the optimal dose of MenSCs, time for checkpoint, patterns of injection, and routes of different diseases should be comprehensively considered in multifaceted studies. For all of the above reasons, we believe that more comprehensive studies are in demand in order to verify the long-term safety and efficacy in MenSC-based treatment.

**Table 1:** The detailed information of MenSCs in the treatment of various diseases

Disease	Donor age	Experimental method	Effect	Reference
DMD	N/A	Murine model, IM, 2 × 10^7^ cells	Differentiation of myogenic cells after 3 W	Cui et al. [25]
POF	N/A	Murine model, IP, 1 × 10^4^ cells	Differentiation of ovarian granulosa cells after 21 D	Liu et al. [36]
T1DM	N/A	Murine model, IV, 3 × 10^5^ cells	Differentiation of β-cells after 14 D	Wu et al. [22]
IUA	24–38	Murine model, Axillary subcutaneous, 1 × 10^6^ cells	Differentiation of endometrial cells after 2 W	Zheng et al. [38]
Sepsis	24–38	Murine model, IP, 2 × 10^6^ cells	Immunomodulation of lower inflammatory responses after 40 H	Alcayaga-Miranda et al. [91]
EC	20–40	Murine model, IV, 1 × 10^6^ cells	Immunomodulation of regulation of B lymphocytes after 10 D	Xu et al. [47]
EC	20–30	Murine model, IV, 1 × 10^6^ cells	Immunomodulation of SDF-1/CXCR4 axis after 10 D	Li et al. [48]
Transplant Vasculopathy	30	Murine model, Aorta, 1 × 10^6^ cells	Immunomodulation of B7-H1 expression after 40 D	Ye et al. [92]
GVHD	18–45	Murine model, IV, 1 × 10^6^ cells	Immunomodulation of lower peripheral blood mononuclear cells after 14 D	Luz-Crawford et al. [45]
IPF	20–40	Murine model, IV, 1 × 10^6^ cells	Immunomodulation of immunosuppressive and antifibrosis effects after 2 W	Zhao et al. [49]
Liver fibrosis	N/A	Murine model, IV, 5 × 10^5^ cells	Paracrine effect of secreting MCP-1, IL-6, HGF, GRO, IL-8, and OPG after 2 W	Chen et al. [18]
MI	N/A	Rat model, IM, 1.5 × 10^6^ cells	Paracrine effect of activate AKT, ERK1/2 and STAT3 after 28 D	Jiang et al. [55]
Stroke	N/A	Rat model, intracerebral, 0.75 × 10^6^ cells	Paracrine effect of secreting VEGF, BDNF, and NT-3 after 14 D	Borlongan et al. [52]
EOC	40	Murine model, SC, 2 × 10^6^ cells	Paracrine effect of promote foxo3a after 28 D	Bu et al. [93]
POF	40	Murine model, IV, 2 × 10^6^ cells	Homing and migration of improving the renewal of germline stem cells after 2 W	Lai et al. [33]
A549-induced tumor	23–42	Murine model, intratumoral, 1 × 10^6^ cells	Homing and migration of target tumor sites after 5 D	Moreno et al. [83]
POF	25–30	Murine model, IV, 2 × 10^6^ cells	Homing and migration of improve the ovarian microenvironment after 21 D	Wang et al. [61]
Cutaneous wound	N/A	Murine model, intradermal, 1 × 10^6^ cells	Immunosuppressive/paracrine effects after 2 W	Cuenca et al. [44]
OCD	25–45	Rabbit model, orthopotic, 7 × 10^5^ cells	Differentiation/Regenerative capacity after 24 W	Khanmohammadi et al. [94]
ALI	N/A	Murine model, IV, 1 × 10^6^ cells	Reduce inflammation/paracrine effect After 72 H	Ren et al. [95]
Spinal cord injury	N/A	Rat model, thoracic, 1 × 10^5^ cells	Reduce inflammation/paracrine effect after 28 D	Wu et al. [53]
MI	N/A	Rat model, IM, 1 × 10^6^ cells	Secreting exosomal microrna-21 after 56 D	Wang et al. [72]
Alzheimer’s disease	N/A	Murine model, hippocampus, 1 × 10^5^ cells	Anti-inflammatory after 7 D	Zhao et al. [96]
POI	25–35	Murine model, IV, 1 × 10^5^ cells	Regulating the ECM-dependent FAK/AKT signaling after 40 D	Feng et al. [97]
POF	N/A	Murine model, IV, 1 × 10^6^ cells	Inhibiting GADD45b expression in the cell cycle after 28 D	Guo et al. [98]
Cervical cancer	N/A	Murine model, subcutaneous, 5 × 10^6^ cells	Mediate TGF-β1-mediated JNK/P21 signaling after 21 D	Liu et al. [99]
HCC	N/A	Murine model, IV, 5 × 10^5^ cells	DNA methylation after 36 D	Wu et al. [78]
CLI	N/A	Rat model, IM, 1 × 10^6^ cells	Secreting growth factors/inhibiting inflammatory responses after 14 D	Murphy et al. [54]
Glioma	N/A	Rat model, Intratumoral, 3 × 10^6^ cells	Inhibition of intracranial glioma growth after 14 D	Han et al. [100]
Multiple sclerosis	18–30	Human model, intrathecal, 6 × 10^6^ cells	Suppress immune responses after 12 M	Zhong et al. [88]
Cardiac fibrosis	N/A	Rat model, IM, 2 × 10^6^ cells	Inhibition of endothelial to mesenchymal transition after 7 D	Zhang et al. [101]
AS	20–40	Human model, IM, 1 × 10^6^ cells	Ensured embryo implantation after 24 M	Tan et al. [89]
ALI	N/A	Murine model, IV, 1 × 10^6^ cells	Downregulation of IL-1 and the upregulation of IL-10 after 48 H	Xiang et al. [59]

*N/A* not applicable, *IM* intramuscular, *IP* intraperitoneal, *IV* intravenous, *SC* subcutaneous, *H* hour, *D* day, *W* week, *M* month, *Ref*. reference, *DMD* Duchenne muscular dystrophy, *CLI* critical limb ischemia, *MI* myocardial infarction, *POF* premature ovarian failure, *T1DM* type 1 diabetes mellitus, *EOC* epithelial ovarian cancer, *GVHD* graft versus host disease, *AS* Asherman’s syndrome, *ALI* acute lung injury, *EC* experimental colitis, *IPF* Idiopathic pulmonary fibrosis, *IUA* intrauterine adhesion, *POI* premature ovarian insufficiency, *OCD* osteochondral defect, *HCC* hepatocellular carcinoma

Ineludible heterogeneity of MenSCs still exists due to donor variability, different processes of cell culture, and various environmental conditions (such as personal operation, injected method, epidemiological background, times, cultural conditions, age, hormonal status, and health status) [19, 34]. These MenSCs are widely applied in preclinical studies and in some clinical research, with many of them displaying effective outcomes to control a variety of diseases. The management of clinical research regarding MenSC-based therapy in diseases will become much more normative and strict. More importantly, some new hotspots are worthy of exploration, such as CRISPR/cas9-mediated gene modification of MenSCs, MenSC-derived exosomes for cell-free therapy, single-cell RNA-seq of MenSCs for precision medicine, engineered MenSCs-based therapy for the delivery platform to enhance the targeting effect, and MenSC niche for improving the microenvironment.

## Conclusion

In summary, although further studies are needed, MenSC-based treatment has great potential for facilitating differentiation, improving immunity, promoting quality, and reducing mortality in various diseases. As MenSCs are a type of adult stem cells having a myriad of therapeutic properties, further elucidation of its mechanism of action is necessary for future clinical applications.

## Data Availability

Please contact corresponding author for data requests.

## Abbreviations

MenSC: Menstrual blood-derived stem cell
CRISPR: Clustered regularly interspaced short palindromic repeats
MSC: Mesenchymal stem cell
BM: Bone marrow
AD: Adipose tissue
UC: Umbilical cord
HLA: Human leukocyte antigen
OCT-4: Octamer binding transcription factor 4
c-kit/CD117: c-kit proto-oncogene
SSEA-4: Stage-specific embryonic antigen-4
iPSC: Induced pluripotent stem cell
DMD: Duchenne muscular dystrophy
POF: Premature ovarian failure
TGF-β: Transforming growth factor-β
PDGF: Platelet-derived growth factor
EGF: Epidermal growth factor
IUA: Intrauterine adhesions
HGF: Hepatocyte growth factor
FGF-4: Fibroblast growth factor-4
OSM: Oncostain M
ALB: Albumin
AFP: α-Fetoprotein
CK: Cytokeratin
CYP 1A1/3A4: Cytochrome P450 1A1/3A4
T1DM: Type 1 diabetes mellitus
DCs: Dendritic cells
NK: Natural killer
Tregs: Regulatory T cells
GVHD: Graft versus host disease
PGE-2: Prostaglandin E-2
PDL-1: Programmed cell death-ligand 1
IDO: Indoleamine 2,3 dioxygenase
IL: Interleukin
CXCR4: cxc chemokin receptor 4
Bcl-2: B cell lymphoma-2
MMP: Matrix metalloproteinases
Bregs: Regulatory B cells
OGD: Oxygen glucose deprivation
BDNF: Brain-derived neurotrophic factor
VEGF: Vascular endothelial growth factor
NT-3: Neurotrophin 3
CLI: Critical limb ischemia
HIF-1α: Hypoxia inducible factor-1 alpha
NO: Nitric oxide
AKT/ERK: Extracellular signal-regulated kinases
STAT 3: Signal transducers and activator of transcription 3
MCP-1: Monocyte chemoattractant protein-1
GRO: Growth-related oncogene
OPG: Osteoprotegerin
SDF-1: Stromal cell-derived factor-1
IGF: Insulin-like growth factor
AS: Asherman’s syndrome
POI: Premature ovarian insufficiency
NF-κB: Nuclear factor-κB
RNA-seq: RNA sequence
OAdv: Oncolytic adenovirus
CRC: Colorectal cancer
